# Anterior Pituitary Volume in Patients with Transfusion Dependent Anemias: Volumetric Approaches and Relation to Pituitary MRI‑R2

**DOI:** 10.1007/s00062-021-01111-4

**Published:** 2021-10-28

**Authors:** Christoph Berliner, Zhiyue J. Wang, Sylvia T. Singer, Regine Grosse, Rosalie V. McDonough, Eric Padua, Qing Yuan, Marcela Weyhmiller, Ellen James, Elliott Vichinsky, Gerhard Adam, Jin Yamamura, Peter Bannas, Roland Fischer, Bjoern P. Schoennagel

**Affiliations:** 1 Department of Diagnostic and Interventional Radiology and Nuclear Medicine, University Medical Center Hamburg-Eppendorf, Martinistr. 52, 20246 Hamburg, Germany; 2 Department of Radiology, University of Texas, Southwestern Medical Center, Dallas, TX USA; 3 Departments of Hematology and Radiology, UCSF Benioff Children’s Hospital, Oakland, CA USA; 4 Department of Neuroradiology, University Medical Center Hamburg-Eppendorf, Hamburg, Germany

**Keywords:** Anterior pituitary gland, Magnetic resonance imaging, Pituitary iron overload, Pituitary R2, Pituitary volumetry

## Abstract

**Purpose:**

Anterior pituitary iron overload and volume shrinkage is common in patients with transfusion-dependent anemia and associated with growth retardation and hypogonadotropic hypogonadism. We investigated the accuracy of different MRI-based pituitary volumetric approaches and the relationship between pituitary volume and MRI-R2, particularly with respect to growth and hypogonadism.

**Methods:**

In 43 patients with transfusion-dependent anemia (12–38 years) and 32 healthy controls (12–72 years), anterior pituitary volume was measured by a sagittal T1 GRE 3D sequence at 1.5T and analyzed by 3D semi-automated threshold volumetry (3D-volumetry). This reference method was compared with planimetric 2D-volumetry, approximate volume calculations, and pituitary height. Using a multiple SE sequence, pituitary iron as MRI-R2 was assessed by fitting proton signal intensities to echo times. Growth and hypogonadism were obtained from height percentile tables and patients’ medical charts. From body surface area and age adjusted anterior pituitary volumes of controls, Z‑scores were calculated for all subjects. Separation of controls and patients with respect to Z and pituitary R2 was performed by bivariate linear discriminant analysis.

**Results:**

Tuned 2D volumes showed highest agreement with reference 3D-volumes (bias −4.8%; 95% CI:−8.8%|−0.7%). A linear discriminant equation of Z = −17.8 + 1.45 **·** R2 revealed optimum threshold sensitivity and specificity of 65% and 100% for discrimination of patients from controls, respectively. Of correctly classified patients 71% and 75% showed hypogonadism and growth retardation, respectively.

**Conclusion:**

Accurate assessment of anterior pituitary size requires 3D or precise 2D volumetry, with shorter analysis time for the latter. Anterior pituitary volume Z‑scores and R2 allow for the identification of patients at risk of pituitary dysfunction.

## Introduction

Endocrine dysfunction from iron accumulation in the anterior pituitary gland is the most common cause of morbidity (> 50%) in patients with transfusion-dependent anemia (TDA), resulting in hypogonadotropic hypogonadism and growth restriction [1–3]. Pituitary gland size and iron deposition using MRI-R2 methods are validated markers of pituitary function [1, 4–6]; however, pituitary size can be assessed by varying metrics, and the accuracy of these methods has not been validated. Furthermore, the relationship between pituitary size, iron accumulation and gland dysfunction is still poorly understood.

In TDA patients, pituitary height assessed from sagittal MR images is commonly used as a surrogate to volumetric measurements due to its simplicity and speed [5, 7–11]; however, pituitary size and shape vary considerably and assessment of pituitary size is subject to a high degree of imprecision unless true volume is measured [12]. It has been postulated that due to its complex 3‑dimensional shape, high-resolution volumetric MRI data would offer more robust metrics of gland health than simple linear height [2]; however, there is a lack of evidence concerning the performance and accuracy of different volumetric approaches, e.g. 2D planimetric models or pituitary height in comparison to the reference standard of pixel-wise 3D analysis.

In addition to providing information on pituitary morphology, MRI can also indirectly measure iron concentration by detecting its paramagnetic properties in pituitary tissue. R2-measurements in the anterior gland have been shown to predict preclinical and biochemical hypogonadism in TDA patients; however, there is paucity of data concerning the relation of pituitary volume and pituitary iron deposition [1, 13, 14]. Similarly, there is limited evidence regarding the relationship of clinical manifestations of pituitary dysfunction (e.g. growth retardation) with pituitary volume and iron accumulation using MRI-R2 relaxometry [15, 16].

The aim of this retrospective study was to determine the accuracy of different approaches for the assessment of anterior pituitary size and the relationship between pituitary size and MRI-R2 relaxometry, particularly with respect to growth retardation and hypogonadism, in patients with TDA.

## Methods

### Study Population

A total of 43 consecutive TDA patients (22 females; mean age 22 years, range: 10–41 years), regularly receiving blood transfusions and treated with iron chelation agents were included in this retrospective study. The underlying diseases were transfusion dependent thalassemia (*n* = 25), Diamond Blackfan anemia (*n* = 7), sickle cell disease (*n* = 6), and rare forms of anemia (sideroblastic, congenital dyserythropoietic and Fanconi anemia). We included 32 controls (19 males; mean age 38 years, range: 12–86 years) without clinical or anamnestic signs of endocrinopathy or hypothalamic region pathology (Table 1) and 13 children (age ≤ 18 years) were included in the patient group and 4 children in the control group.

**Table 1:** Characteristics of transfusion dependent anemia (TDA) patients and controls: median and interquartile range (IQR) of age, body surface area (BSA), pituitary height, 3D volume (V3D) and 2D volume (V2D_tuned_), and body height percentiles; Mann-Whitney U‑test between groups, and Spearman rank correlation (R_S_, *p*) of pituitary R2 with other parameters (R2xParam.)

Parameter	TDA patients			Controls			U‑test	R2xparam
	*n*	*Median*	*IQR*	*n*	*Median*	*IQR*	*p*	*R*_*S*_*, p*
**Children (≤** **18 years)**	13	–	–	4	–	–	–	–
**Age (years)**	43	20	8	32	35	25	< 10^−5^	0.10, 0.4
**BSA (m**^**2**^**)**	43	1.5	0.2	32	1.8	0.3	< 10^−6^	−0.18, 0.2
**Pituitary R2 (s**^**−1**^**)**	43	13	5	22	10	1	< 10^−4^	1.0, 0
**Pituitary height (mm)**	43	5.5	1.4	32	7.0	1.0	< 10^−6^	−0.53, 5 ∙ 10^−6^
**V3D (mm**^**3**^**)**	43	349	197	30	527	124	< 10^−3^	−0.55, 3 ∙ 10^−6^
**V3D**_**males**_** (mm**^**3**^**)**	21	385	158	18	494	128	2 ∙ 10^−2^	−0.47, 6 ∙ 10^−3^
**V3D**_**females**_** (mm**^**3**^**)**	22	319	211	12	571	115	< 10^−2^	−0.65, 10^−4^
**V2D**_**tuned**_** (mm**^**3**^**)**	33	346	141	25	541	108	< 10^−3^	−0.45, 10^−3^
**Body height (%)**	43	21	40	32	70	32	< 10^−5^	−0.19, 0.1

Body height and weight was measured for each individual at the time of MRI assessment to calculate body surface area (BSA) and growth percentiles. The latter was calculated from height, gender, age, and ethnicity using the World Health Organization (WHO) (https://www.omnicalculator.com/health/child-height-percentile) or the Centers for Disease Control and Prevention (CDC) (https://tall.life/height-percentile-calculator-age-country) standard.

3‑dimensional volumes (V3D) and pituitary R2 were available for all patients and assessed within 0–3 months (median: same day) of the MRI measurement of the pituitary gland (Table 1). Diagnosis of hypogonadism was obtained from medical charts. Diagnostic criteria involved lack of secondary puberty, amenorrhea in females or need of testosterone replacement in males. Females or males between 10–14y with constantly low gonadotropin and estradiol/testosterone levels were also diagnosed for hypogonadism.

Patients and controls were measured between 2006–2017 at the radiology departments of two university medical centers in Europe (*n* = 40) and the USA (*n* = 35). All procedures were approved by the institutional committees on human research at both centers. Informed written consent was obtained from all individuals.

### MR Imaging Protocol

MRI of the pituitary gland was performed using an 8‑element head coil on two different 1.5T MRI systems (Symphony®, Siemens Healthcare, Erlangen, Germany; Intera®, Philips Healthcare, Best, The Netherlands).

For Siemens/Philips systems and volume assessment, a gradient-recalled echo T1 weighted 3D sequence in sagittal orientation was applied with 1 mm isotropic voxels (TR = 17/22 ms, TE = 3.7/4.6 ms, FA = 12°/30°, matrix = 256 × 256, pixel size 1.0 × 1.0 mm^2^, 100 slices, slice thickness = 1 mm, gap = 0 mm, bandwidth = 210/47 Hz/*p*x). Pituitary R2 was assessed using a multi-echo spin-echo sequence in sagittal orientation (Siemens/Philips: TR = 2500 ms, TE1 = 15 ms, ΔTE = 15 ms, echo train length 8, matrix = 256 × 256, pixel size 0.78 × 0.78 mm^2^, slices = 11, slice thickness = 3 mm, gap = 0.3 mm, bandwidth = 95/120 Hz/*p*x). Total data acquisition time was approximately 30 min. The MRI protocol was implemented by one experienced radiologist (Z.J.W.) at both institutions.

A monoexponential function for proton signal intensities and echo time (including all 8 echoes) was used for determination of the relaxation rate R2 with no signal level off-set using an in-house software (IDL 7.0). The anterior pituitary ROI was manually drawn along the boundary of the gland from 3–4 slices. Agreement between R2 from pixel-wise and ROI-wise analysis was −0.07 ± 0.04 s^−1^ (95% CI: −0.15 s^−1^–0.02 s^−1^).

### Pituitary Volumetry

#### Semi-automated Threshold 3D Volumetry (V3D)

**Fig. 1:**
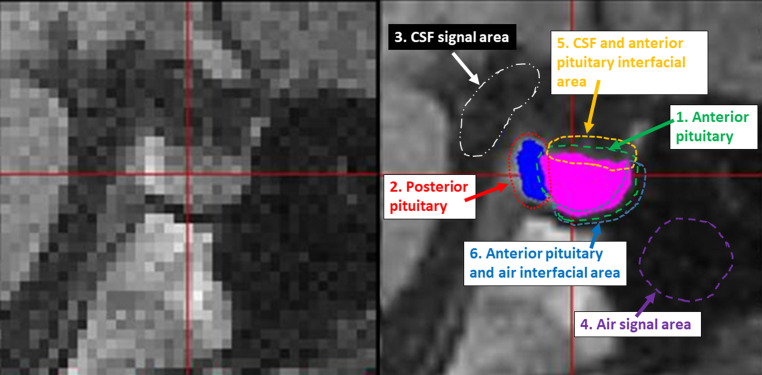
Demonstration of semi-automatic threshold in V3D anterior pituitary volumetry. The *left* shows a T1-weighted mid-sagittal MR image of the pituitary gland. On the *right*, the image is 6‑fold interpolated, and manually drawn ROIs used for generating the anterior pituitary mask are shown. ROIs of anterior (1) and posterior (2) pituitary were sufficiently eroded to remove the exterior signal background before the interior signal was averaged. The signals for CSF (ROI 3), and air space (ROI 4) were directly averaged. The boundary between the anterior pituitary and CSF was determined in ROI 5 by setting the threshold to the average of CSF mean value and anterior pituitary mean value. The boundary between the anterior pituitary and air space was determined in ROI 6 by setting the threshold to the average of air space mean value and anterior pituitary mean value. The boundary between the anterior and posterior pituitary was determined inside the overlapping region of ROIs 1 and 2, with the threshold equal to the average signal intensity of the two glands. The *solid pink and blue areas* represent the final masks used for anterior and posterior pituitary volume calculation

The approach combined two methods for determination of the boundary of the anterior pituitary gland. Firstly, the ROI information was provided in two slice orientations (sagittal and axial reconstructions). Manual tracing was performed using multiple sagittal and axial planes. The computer routine combines the tracing to obtain a 3D ROI. This two-plane tracing approach was used because different boundaries were best visualized on different orientations. For example, the boundary of the left and right side of the pituitary gland from the adjacent cavernous sinus was easy to visualize on the axial plane but difficult on the sagittal plane. Second, the boundaries of the anterior and posterior pituitary glands were determined by setting thresholds in interfacial areas with surrounding structures (Fig. 1). Anterior pituitary volumes were then obtained. One data set can be processed in approximately 20 min. Details of the software have been previously described [17].

#### Planimetric 2D Volumetry (V2D)

MRI 3D data sets of the pituitary were imported into an external analysis software (Volume Viewer, GE-Healthcare, Chicago, IL, USA). The external contours of the anterior pituitary gland were manually delineated in all axial slices covering the anterior pituitary (≤ 10 slices). The posterior pituitary gland was differentiated from the anterior gland by its brighter signal intensity. The volume was obtained by adding the areas of each slice. Volumes were independently assessed by two operators (CB and BS) to determine interoperator variability (V2D_CB_ and V2D_BS_).

In a more complex step, delineation of pituitary contours was controlled and eventually corrected in all 3 planes (axial, coronal, sagittal) resulting in a fine-tuned 2D volume (V2D_tuned_). One data set was processed in about 5–10 min.

#### Approximate Volumetry (V_ROI_, V_Ell_, and V_AL_)

Approximate assessment of anterior pituitary volumes was based on the classical formula for the volume of an ellipsoid, V = 4π/3 **·** a **·** b **·** c, with axes a, b, and c. The fine-tuned V2D_tuned_ procedure also allowed the determination of several pituitary ROIs and diameters in different planes: ROI_trans_, ROI_cor_, and left-to-right (d_lr_), anterior-to-posterior (d_ap_), and feet-to-head (d_fh_) diameter. The following approximate volumes of the anterior pituitary gland were calculated as

$$\mathrm{V}_{\mathrm{ROI}}=8/3\pi \cdot \mathrm{ROI}_{\text{trans}}\cdot \mathrm{ROI}_{\mathrm{cor}}/\mathrm{d}_{\mathrm{lr}},$$

$$\mathrm{V}_{\mathrm{Ell}}=\pi /6\cdot \mathrm{d}_{\mathrm{ap}}\cdot \mathrm{d}_{\mathrm{fh}}\cdot \mathrm{d}_{\mathrm{lr}},$$

and by the area-length method V_AL_ = 2/3 **·** ROI_trans_ / d_fh_.

The most straightforward information about the size of the anterior pituitary is the maximum pituitary height measured in mid-sagittal images as previously described [18].

### Statistics

All values are presented as median values with interquartile ranges (IQR). Variables between groups were tested for significance using the nonparametric Wilcoxon-Mann-Whitney U‑test. *P*-values < 0.05 were considered statistically significant. Pituitary R2 was compared with other parameters using Spearman-rank correlation (r_S_). Comparison of volumetric approaches was performed by relative difference analysis versus the reference method of V3D. The interoperator variability was assessed by Bland-Altman analysis.

Multiple regression analysis (forward and backward stepwise) was used for prediction, characterized by the coefficient of determination (r^2^). Normal BSA-adjusted pituitary 3D volumes (V3D_i_) were calculated from controls as a function of age, subtracted from measured V3D_i_, and expressed as Z‑values. Separation of controls and TDA patients with respect to Z and pituitary R2 was performed by bivariate linear discriminant analysis with *a priori equal group size*, which brings different patient (*n* = 43) and control numbers (*n* = 32) to the same level (STATISTICA 6.1, Stat Soft. Inc., Tulsa, OK, USA).

## Results

Transfusion dependent anemia (TDA) patients were significantly younger, had significantly lower BSA, pituitary height, and volume (V3D), but higher anterior pituitary R2 values compared to controls (*p* < 10^−4^). Significant correlations with pituitary R2 were found for pituitary height and volume (Table 1).

### Pituitary Volume Data Analysis

Pituitary height was assessed in all controls (*n* = 32) and TDA patients (*n* = 43). Pituitary planimetry (V2D_tuned_) and volumetric estimations by V_ROI_, V_Ell_, and V_AL_ were assessed in 58 of 75 patients and controls (77%). Comparison of interoperator variability was performed by V2D_CB_ and V2D_BS_ and in 48 subjects. Reference 3‑dimensional volume (V3D) was assessed in 42 patients and 30 controls (*n* = 72, 96%). The missing V3D volumes were due to artifacts or incomplete 3D-data sets.

**Fig. 2:**
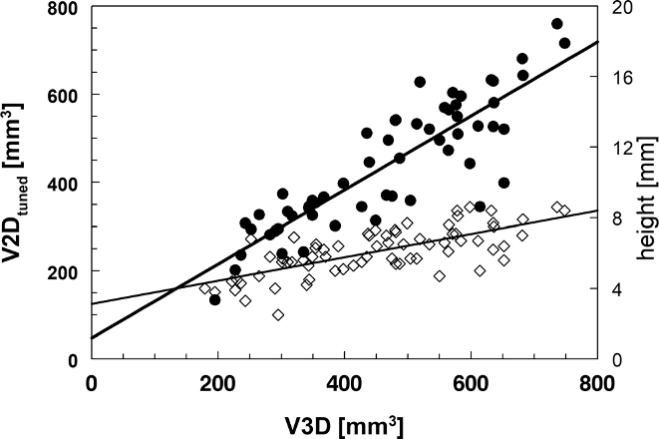
Significant correlation between pituitary volumetric methods V3D and V2D_tuned_ (*solid circles*, r_S_ = 0.86) or pituitary height (*rhomboids*, r_S_ = 0.64, right scale) in all subjects

V3D as the volumetric standard of reference was compared to all other pituitary measures by Pearson correlation and relative difference (Altman-Bland) tests. In Fig. 2, exemplary significant correlations (both *p* < 0.0001) of the anterior pituitary volumes V3D with V2D_tuned_ (R_S_ = 0.86) and pituitary height (R_S_ = 0.64) are shown.

Relative differences between the reference volume V3D and other volumetric estimators are shown in Table 2. The 2‑dimensional method V2D_tuned_ showed the highest agreement with the reference volume, with a mean deviation of −4.8% (95%CI: −8.8%|−0.7%). Comparison of V2D_CB_ with V_ROI_ and V_Ell_ revealed mean deviations of −9.0% and −10.0%, respectively (data not shown).

Interoperator agreement for V2D between the two operators (V2D_CB_ and V2D_BS_) by Bland-Altman test was good (bias 2.6% ± SE 1.7%) (Table 2).

**Table 2:** Pituitary volumes assessed by MRI: comparison of reference V3D and other approximate volumetric methods (*p* < 10^−4^), numbers indicate relative differences with ±95% lower and upper limit of agreement (LoA, UpA)

Methods	*n*	R_S_ ^a^	Bias (%)	±SE (%)	−95% LoA (%)	+95% UpA (%)	±SE (%)
**V3DxV2D**_**tuned**_	58	0.86	−4.8	2.0	−35.0	25.4	3.5
**V3DxV2D**_**CB**_	49	0.72	−14.9	3.0	−56.6	26.8	5.2
**V3DxV2D**_**BS**_	48	0.73	−13.3	3.0	−54.4	27.9	5.2
**V3DxV**_**ROI**_	58	0.69	−24.3	2.9	−68.2	19.5	5.1
**V3DxV**_**Ell**_	58	0.63	−21.0	3.2	−69.5	27.5	5.6
**V3DxV**_**AL**_	58	0.75	−18.7	2.8	−60.6	23.4	4.8
**V2D**_**CB**_**xV2D**_**BS**_ ^b^	48	0.95	2.6	1.7	−20.8	26.0	3.0

*V3D* semi-automated threshold 3D volumetry, *V2D*_*tuned*_ planimetric 2D volumetry with corrections, *V2D*_*CB*_ planimetric 2D volumetry by operator CB, *V2D*_*BS*_ planimetric 2D volumetry by operator BS, *V*_*ROI*_ approximate volumetry by region of interest, *V*_*Ell*_ approximate volumetry by ellipsoid model, *V*_*AL*_ approximate volumetry by area-length model

^a^ Spearman rank correlation coefficient

^b^ Bland-Altman test

### BSA-adjusted Pituitary Volume and Age in Controls

For controls, multiple regression of pituitary volume (V3D) with age, BSA, and R2 resulted in significant contributions only from age (*p* = 0.01), in contrast to the negative univariate regression with age V3D(mm^3^) = 604–2.3 · age (r^2^ = 0.11, *p* = 0.04).

**Fig. 3:**
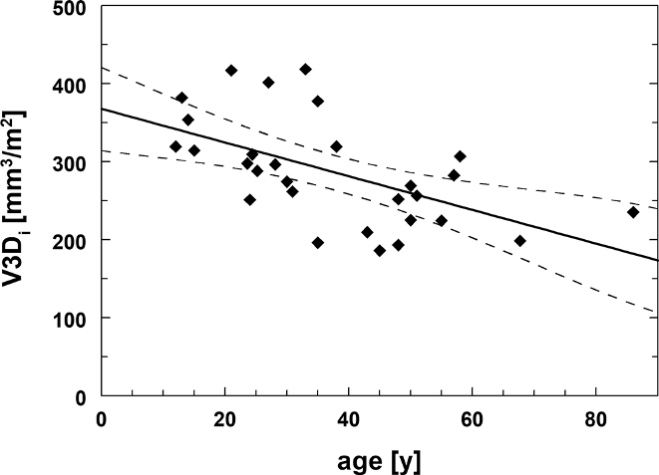
Relationship between BSA adjusted V3D (=V3D_i_) and age in controls (dashed lines indicate 95% confidence interval, r^2^ = 0.29)

This relationship with age significantly improved for the BSA-adjusted volume V3D_i_ (*p* = 0.002, r^2^ = 0.29) and resulted in a linear relationship of V3D_i_ = a0 + a1 · age with a0 = 368 ± 25mm^3^/m^2^, a1 = −2.16 ± 0.61mm^3^/m^2^/year, and a standard error of estimate (SEE) = 57 mm^3^/m^2^ (Fig. 3). The obtained equation allows calculation of anterior pituitary reference volumes as function of age (V3D_i_(age)) from controls and thus Z‑scores for all subject volumes (V3D_i_) by Z = (V3D_i_ − V3D_i_(age)) / SEE.

### Discriminant Analysis of Patients from Controls by Z‑scores and R2

Since age, R2, and BSA are more strongly associated with V3D of patients compared to controls, multiple regression analysis was applied to all subjects resulting in significant contributions from BSA (*p* = 0.0003), R2 (*p* = 0.0005), and age (*p* = 0.03). R2 (*p* = 0.0002) and age (*p* = 0.04) were significantly associated with V3D/BSA (V3D_i_). Similarly, for BSA adjusted pituitary height, we obtained significant associations from R2 (*p* = 0.001) and age (*p* = 0.03), in contrast to V2D/BSA, where only R2 contributed significantly (*p* = 0.01).

**Fig. 4:**
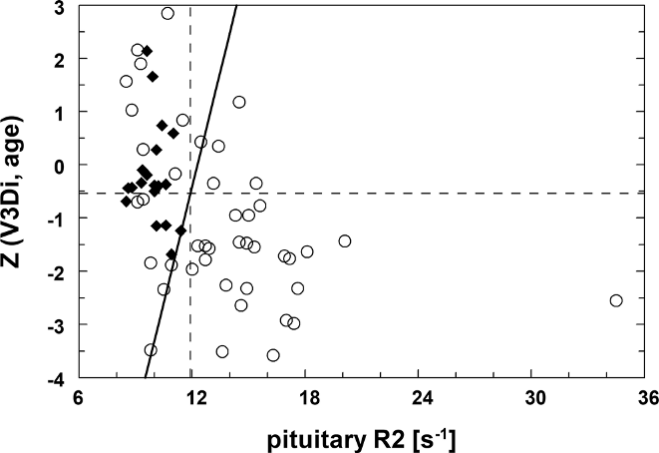
Z‑values calculated from BSA and age adjusted pituitary 3D-volumes (Z(V3D_i_, age)) are associated with pituitary relaxation rate R2. A linear discriminant function (*solid line*, *p* < 0.0016) separates controls (*rhomboids*) from transfusion dependent patients (*circles*). Univariate thresholds for R2 and Z are indicated by *dashed lines* at 11.9 s^−1^ and −0.54, respectively

Using age and BSA adjusted V3D Z‑scores Z(V3D_i_, age) for pituitary volume in patients (*n* = 43) and controls (*n* = 19) allowed separation of the two groups by bivariate discriminant analysis (Z, R2) along a linear discriminant (cut-off) line of Z = −17.8 + 1.45 **·** R2 (Fig. 4). A sensitivity and specificity of 65% (28/43) and 100% (19/19) was obtained for correctly classifying patients and controls, respectively. Univariate discriminant analysis resulted in thresholds of Z(V3D_i_, age) = −0.54 or R2 = 11.9 s^−1^ with specificities of 76% or 100%, respectively, and similar sensitivities (70% or 67%). For bivariate age and BSA adjusted V2D Z‑scores Z(V2D_i_, age) versus R2, discriminant analysis in only 42 patients and controls showed a sensitivity of 61% and a specificity of 93% (data not shown).

Growth was assessed as height percentiles with median values (IQR) for patients and controls of 21% (9–49%) and 70% (53–85%), respectively (Table 1). Univariate discriminant analysis based on growth significantly separated patients and controls at 47.2% (sensitivity 70% and specificity 78%). In the 28 patients correctly classified by the linear discriminant equation of Fig. 4, growth was reduced in 21 (IQR = 9%–29%) of them with a sensitivity of 75%, while 7 patients (IQR = 57%–80%) were in the range of controls (IQR = 54%–82%) (data not shown).

**Fig. 5:**
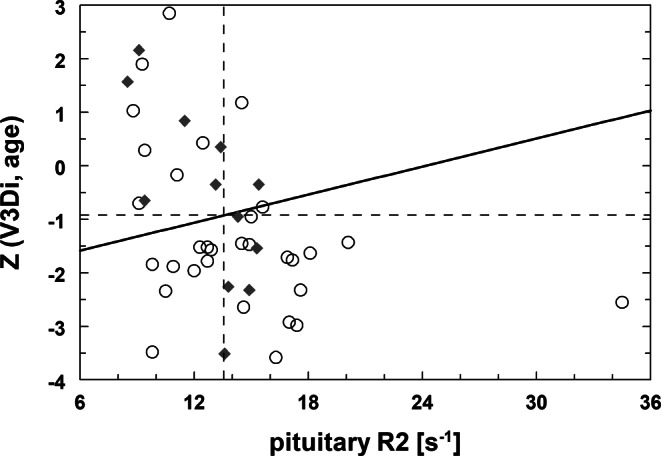
Z‑values calculated from BSA and age adjusted pituitary 3D-volumes (Z(V3D_i_, age)) are associated with pituitary relaxation rate R2. A linear discriminant function (*solid line*, *p* < 0.0016) separates patients with hypogonadism (*circles*) and without hypogonadism (*rhomboids*). Univariate thresholds for R2 and Z are indicated by *dashed lines* at 13.6 s^−1^ and −0.92, respectively

Hypogonadism was reported in 31/42 patients. Using a bivariate discriminant analysis (Z(V3D_i_, age), R2) along a linear discriminant (cut-off) line of Z = −2.11 + 0.087 **·** R2 allowed separation of patients with and without hypogonadism (Fig. 5). A sensitivity of 74% was obtained for correctly classifying patients with hypogonadism. The same sensitivity could be achieved for V3D/BSA alone at a threshold of 270 mm^3^/m^2^, while R2 alone was relatively insensitive for hypogonadism (48%). For univariate V2D Z‑scores Z(V2D_i_, age), a sensitivity of 73% at a threshold of 275 mm^3^/m^2^ was calculated for 26/33 patients with hypogonadism.

## Discussion

This MRI study in patients with TDA evaluated the accuracy of different geometric approaches for assessing the volume of the anterior pituitary gland. It also investigated the association of pituitary size, growth retardation, and hypogonadism, as an indicator of pituitary dysfunction, with iron levels in the pituitary gland using MRI-R2 relaxometry.

Compared to the reference standard of semi-automated threshold-dependent V3D, V2D planimetry revealed the highest agreement and was superior to approximated volumetry (V_ROI_, V_Ell_, and V_AL_) and pituitary height. Z‑scores of pituitary volume were associated with R2 rates and revealed threshold-derived specificities of 76% and 100% for both parameters to discriminate patients from controls, respectively. In 75% and 71% of patients correctly classified by volume Z‑scores and R2 rates, growth restriction (defined below the 47th percentile) and hypogonadism was present, respectively.

As pituitary size is an independent predictor of preclinical hypogonadism in adult patients, reliable measurement of the anterior pituitary volume is critical [1]. Few studies have performed true planimetry (V2D) [12, 19, 20] or indirect volume estimations (V_ROI_ and V_ell_) [12, 21, 22] and, to our knowledge, even fewer employed a more sophisticated semi-automated V3D approach [23]. In this study, volumetric comparisons using V3D as the reference method revealed highest accuracy for V2D planimetry (V2D_tuned_) with a mean deviation of only −5%. Conversely, indirect volume estimates (V_ROI_, V_Ell_, V_AL_) revealed significant bias of up to −24% and appear insufficient to reflect true pituitary volumes. Our results confirm the hypothesis that size assessment is subject to a high degree of imprecision unless the true volume is measured [12]. Considering the long post-processing times of around 20 min and the dedicated expertise and software required for V3D, we recommend true planimetry (V2D_tuned_) for accurate calculation of pituitary gland volumes. The required 3D image data acquisitions and an approximated post-processing time of 5 min are assumed worthwhile, considering the merit of precise volumetric results. The good interoperator agreement for V2D (bias below 3%) emphasizes its clinical utility in daily routine.

BSA-adjusted anterior pituitary volumes of healthy controls were in agreement with reported normal values [20] considering the older age of our controls. There is a well-known relation of anterior pituitary volume with age, revealing a typical increase before and during puberty [6, 12], followed by a plateau phase or slight decline of volume starting in the 3rd decade of life [8, 20, 21]. Our study using V3D also revealed a decrease in anterior pituitary volume with age and encompassed a broader age spectrum than former studies which focused on the first and second decades of life [10, 12, 19, 24]. Our study revealed lower pituitary volumes (mean = 516 mm^3^) for controls than a study in 94 women (age 18–90 years) [25]; however, the inverse relationship with age could be confirmed if volumes were adjusted for these lower values within limits.

Anterior pituitary volume Z‑scores (adjusted for BSA and age) were associated with pituitary R2 rates. Combined volume Z‑scores and R2 allowed reliable discrimination of patients from controls with a sensitivity and specificity of 65% and 100%, respectively. From univariate analysis, R2 was superior to discriminate patients from controls than volumetric Z‑scores. Pituitary size can be affected by different factors, but in patients with TDA pituitary shrinkage is primarily thought to originate from iron deposition [1, 2, 7, 13]. The fact that volumetric Z‑scores were significantly dependent on R2 supports the idea of pituitary iron deposition to be the causative mechanism for developing pituitary volume loss.

Hypogonadism and growth retardation are frequent endocrine complications associated with the anterior pituitary gland in TDA patients, with a reported prevalence of 50–80% [26, 27]; however, there are only few studies investigating the association of pituitary iron and endocrine function. In our cohort of TDA patients, growth restriction and hypogonadism were clearly linked to pituitary R2 and volume loss. In patients who were correctly identified by abnormal pituitary volume Z‑scores and R2, growth retardation defined by the 47th percentile and hypogonadism were a frequent finding (75% and 71%). This is comparable to observations where pituitary size and excess iron were independently associated with hypogonadism [1, 4, 5, 13]. In addition, our results showed that precise volume assessment seems to be more important than R2 to predict hypogonadism.

Although R2 is an independent biomarker of pituitary function, the causal relationship with pituitary size is not fully understood and studies to analyze this relation are rare [1, 4]. This study supports the finding that pituitary iron deposition reflected by R2 leads to pituitary volume loss and subsequent gland dysfunction, as corroborated by the high rate of growth retardation in our patient group.

The etiology of pituitary dysfunction is complex but is mainly attributed to excess iron depositions [13, 15], with the anterior lobe being particularly prone to the toxic effects of iron overload [28]; however, factors predicting the development of pituitary iron overload are not well understood [1, 11].

A potential limitation of this study is the lack of endocrine measures (e.g. hormone stimulating tests); however, these tests have been described as being imprecise and suffer from poor reproducibility [29]. We also did not investigate the impact of estrogen/testosterone on pituitary size since many patients were under replacement therapy. Although MR imagers from two manufacturers were used, no significant differences were observed between Siemens/Philips for R2 (*n* = 34/31, *p* = 0.4) or V3D (*n* = 34/39, *p* = 0.2).

Other limitations like missing age and gender matched controls were due to the retrospective character of the study; however, the utilization of Z‑scores usually compensates for this drawback. With respect to Z‑scores a more uniform stratification of age would have been beneficial.

## Conclusion

Accurate assessment of pituitary size requires true V3D or alternatively modified 2D planimetry (V2D_tuned_), with preference of the latter due to shorter analysis time and no need for specialized software. Pituitary volume and R2 allow the discrimination of TDA patients from healthy individuals. Abnormal values are frequently associated with growth retardation, and hypogonadism may be preferentially predicted by volume shrinkage. In the future, assessment of pituitary volume and R2 may help to detect gland damage before endocrine dysfunction becomes overt. Further research is necessary to better understand the relation of pituitary volume, R2 and endocrine dysfunction relative to age.
